# Causes of Mortality in Korean Patients with Neurodegenerative Dementia

**DOI:** 10.1155/2022/3206594

**Published:** 2022-04-25

**Authors:** Hyo Geun Choi, Bumjung Park, Ji Hee Kim, Joo-Hee Kim, Mi Jung Kwon, Miyoung Kim

**Affiliations:** ^1^Hallym Data Science Laboratory, Hallym University College of Medicine, Anyang, Republic of Korea; ^2^Department of Otorhinolaryngology-Head & Neck Surgery, Hallym University Sacred Heart Hospital, Hallym University College of Medicine, Anyang, Republic of Korea; ^3^Department of Neurosurgery, Hallym University Sacred Heart Hospital, Hallym University College of Medicine, Anyang, Republic of Korea; ^4^Division of Pulmonary, Allergy, And Critical Care medicine, Department of Medicine, Hallym University Sacred Heart Hospital, Hallym University College of Medicine, Anyang, Republic of Korea; ^5^Department of Pathology, Hallym University Sacred Heart Hospital, Hallym University College of Medicine, Anyang, Republic of Korea; ^6^Department of Laboratory Medicine, Asan Medical Center, University of Ulsan College of Medicine, 88 Olympic-ro 43-gil, Songpa-gu, Seoul 05505, Republic of Korea

## Abstract

The prevalence of neurodegenerative dementia is increasing owing to the rapid growth of the older population. We investigated risks and causes of mortality in Korean patients with neurodegenerative dementia using data from the Korean Health Insurance Review and Assessment Service-National Sample Cohort with the aim to improve their care. From a pool of 1,125,691 patients, 11,215 patients aged ≥60 years who were diagnosed with dementia between 2002 and 2013 were examined along with 44,860 matched controls. A Cox proportional hazard model was used to calculate crude and adjusted hazard ratios (HRs). During the follow-up period, 34.5% and 18.8% of individuals in the neurodegenerative dementia and control groups, respectively, died (*P* < 0.001). The adjusted HR for mortality in the neurodegenerative dementia group was 2.11 (2.41 and 1.96 in men and women, respectively). Moreover, the adjusted HRs in patients with neurodegenerative dementia were 3.25, 2.77, and 1.84 for those diagnosed at ages 60–69, 70–79, and ≥80 years, respectively. The highest odds ratio for mortality was noted among patients with neurologic disease (15.93) followed by those with mental disease (4.89). These data show that the risk of mortality increased regardless of age and sex in Korean patients with neurodegenerative dementia.

## 1. Introduction

Dementia is the loss of function in one or more cognitive domains to an extent severe enough to interfere with independent social or occupational activities [1, 2]. It is commonly associated with more than one neuropathological disorder, usually Alzheimer's disease (AD) with cerebrovascular pathology [1, 2]. As the global proportion of the older population has grown in recent decades, the prevalence of dementia has also increased rapidly [3]. The Global Burden of Diseases, Injuries, and Risk Factors study of 2016 revealed that the rate of this condition increased from 383 per 100,000 population in 1990 to 593 per 100,000 population in 2016 (a 54.7% jump) [3]. The number of individuals who live with dementia worldwide increased from 20.2 million in 1990 to 43.8 million in 2016 [3]. Indeed, the prevalence of dementia is expected to increase in the coming years, as is its impact on society (including the burden imposed on caregivers) [4, 5].

Dementia not only worsens patients' quality of life but may also subject them to a higher risk of death. Thus, understanding the risks and causes of mortality among individuals with dementia is important for patients, caregivers, health professionals, and health policymakers [6]. Previous Western studies consistently showed that the life expectancy of older people with dementia is shorter than that of their healthy counterparts [6–8].

The growth rate of the older population in Korea is one of the highest in the world [9]. The nation has been projected to become a “super-aged” society by 2026, with 37.6% of the population (17.9 million individuals) expected to be over 65 years of age by 2050 [9]. As the society has aged, the prevalence of dementia has also increased rapidly in Korea [9, 10]. A meta-analysis published in 2014 found that the prevalence of dementia in patients aged ≥65 years was 9.2%, which was higher than that in similarly aged counterparts in Western and other Asian countries [9]. Studies on this subject in Korea are scarce and have yielded conflicting results. A study of 724 Korean patients with AD showed that the median survival was 9.3 years, which was much longer than that found in studies of Caucasians [11]. However, results from a more recent study suggested that the risk of mortality in Korean patients with dementia is underestimated as the hazard ratio (HR) of individuals who had dementia at both the baseline and 2-year follow-up assessment was 2.82, while that of individuals who had no dementia at baseline assessment but had developed the disease by the time of the 2-year follow-up visit was 8.37 [12].

The risks and causes of mortality in patients with dementia could be ethnicity and environment specific; thus, reliable data from a representative Korean population with this disease are needed to better understand these aspects and assist patients with dementia in this country. As such, we performed this retrospective large-scale longitudinal follow-up study of risks and causes of mortality in patients with dementia in South Korea. Using a national cohort database, we could analyze a representative population over a maximum follow-up period of 12 years.

## 2. Materials and Methods

### 2.1. Study Population and Data Collection

The ethics committee of Hallym University approved the use of these data (2019-01-003); the requirement for written informed consent was waived by the Institutional Review Board. All methods were performed in accordance with the relevant guidelines as well as the tenants laid down in the Declaration of Helsinki. This national cohort study relied on data from the Korean Health Insurance Review and Assessment Service-National Sample Cohort [13, 14].

### 2.2. Participant Selection

Among 1,125,691 patients with 114,369,638 medical claim codes, we included 13,102 participants who were diagnosed with neurodegenerative dementia between 2002 and 2013. Neurodegenerative dementia included AD (International Classification of Diseases- (ICD-) 10 code G30) or dementia in AD (F00). For accurate diagnosis, we selected participants who were treated 2 or more times for neurodegenerative dementia; the reliability of the diagnosis was as described in our previous studies [15, 16]. The control participants were identified from among 1,112,589 individuals who had not been diagnosed with neurodegenerative dementia between 2002 and 2013.

Participants with neurodegenerative dementia were compared to control participants who were matched 1 : 4 for age, sex, income, region of residence, and past medical histories of hypertension, diabetes mellitus, and dyslipidemia. To prevent selection bias, control participants were each assigned a random number and then selected in descending numerical order. The index date was set as that of the diagnosis of dementia; participants in the control group were followed from the same index date as their matched counterparts. The follow-up duration was calculated from the index date to the date of death or that of the end of the study (December 31, 2013), and the participants were followed until death or were otherwise censored. Individuals in the control group who died before their matched counterparts in the neurodegenerative dementia group entered the study were excluded. The cause of death was defined according to the death certificate issued by medical doctors, which stated the most relevant reason for death. These data were obtained from Statistics Korea (http://kostat.go.kr/portal/eng/index.action).

**Figure 1:**
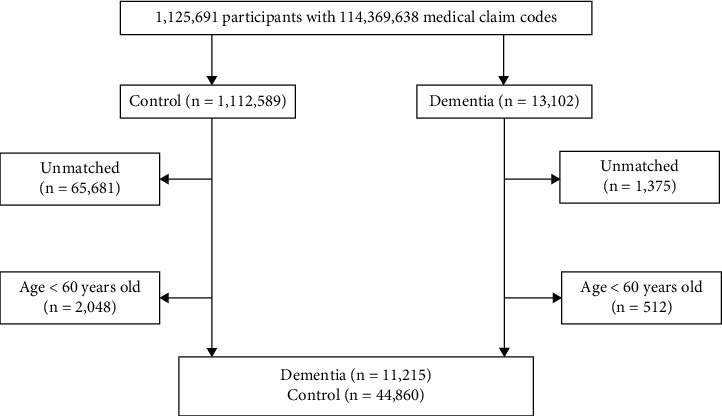
A schematic illustration of the participant selection process that was used in the present study. Of a total of 1,125,691 participants, 13,102 with neurodegenerative dementia were included; these patients were matched 1 : 4 with a control group of individuals not diagnosed with dementia. Ultimately, 11,215 participants with neurodegenerative dementia and 44,860 controls were included.

Participants with neurodegenerative dementia for whom we could not identify a sufficient number of matching control individuals were excluded (*n* = 1,375), as were individuals who were diagnosed with neurodegenerative dementia before the age of 60 years (*n* = 512). Ultimately, 1 : 4 matching resulted in the inclusion of 11,215 individuals with dementia and 44,860 control participants (Figure 1); however, they were not matched for past medical histories of ischemic heart disease and stroke, as these events were relatively rare.

### 2.3. Variables

Each participant's age, sex, income, and region of residence were noted as described in our previous study [17]. The cause of death was determined according to the Korean standard classification of diseases, as also described in that same study [17].

The medical histories of the participants were evaluated using ICD-10 codes. For the accuracy of diagnosis, a patient was considered to have hypertension (I10 and I15), diabetes (E10–E49), and hyperlipidemia (E78) if treated 2 or more times for these conditions and was considered to have ischemic heart disease (I24 and I25) and stroke (I60–I66) if treated 1 or more times for either.

### 2.4. Statistical Analyses

The chi-square or Fisher's exact test was used to compare mortality rates between individuals in the neurodegenerative dementia and control groups according to the cause of death. The false discovery rate was calculated to adjust for an incorrectly rejected null hypothesis.

The Cox proportional hazard model was used to calculate the HR for mortality in patients with neurodegenerative dementia. In this analysis, both a crude (simple) model and another adjusted for histories of ischemic heart disease and stroke were used; the 95% confidence intervals (CIs) were also calculated. Patients were stratified according to age, sex, income, region of residence, hypertension, diabetes, and hyperlipidemia status. Two-tailed analyses were conducted, and *P* values of < 0.05 were considered statistically significant. The results were analyzed using the SPSS software version 21.0 (IBM, Armonk, NY, USA).

## 3. Results

**Figure 2:**
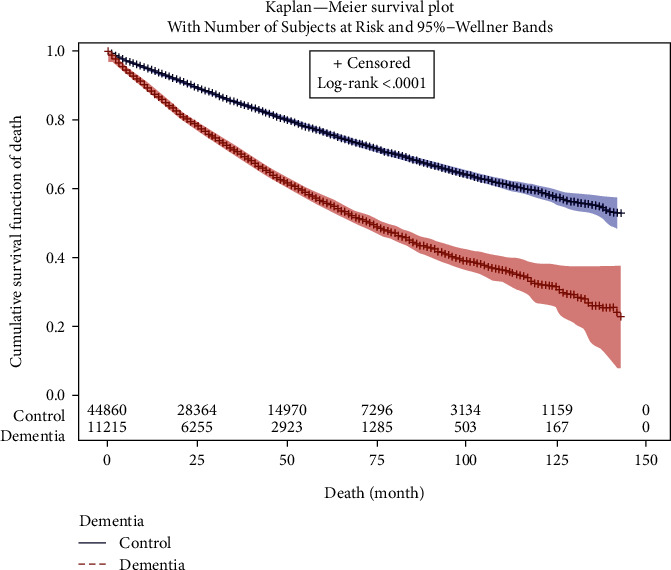
Kaplan–Meier curves showing mortality rates of Korean patients with dementia versus those of controls over the duration of the study.

The mean follow-up durations were 35.9 months (standard deviation (SD) = 29.6 months) in the neurodegenerative dementia group and 41.9 months (SD = 32.4 months) in the control group. The survival rates during the study period are shown in Figure 2. Age, sex, income level, and region of residence were matched between the neurodegenerative dementia and control groups (Table 1). The incidence rates of hypertension, diabetes, and dyslipidemia did not differ between the 2 groups, whereas ischemic heart disease and stroke were more prevalent in the neurodegenerative dementia group. Furthermore, 34.5% (3,873/18,557) and 18.8% (8,434/44,860) of participants in the neurodegenerative dementia and control groups, respectively, died during the follow-up period (*P* < 0.001).

The crude and adjusted HRs for mortality in the neurodegenerative dementia group were 2.26 (95% CI = 2.17–2.35) and 2.11 (95% CI = 2.03–2.20), respectively (Table 2). This group had higher crude and adjusted HRs for mortality across all age- and sex-based subgroups than the control group (Table 2). The adjusted HR (95% CI) was the highest in patients with neurodegenerative dementia whose ages at the initial diagnosis were 60–69 years (3.25 [2.79–3.78]), which was lower in those aged 70–79 years (2.48 [2.32–2.65]), and lowest in those aged ≥80 years (1.78 [1.69–1.88]). The adjusted HRs (95% CIs) were 2.41 (2.26–2.57) and 1.96 (1.86–2.06) in men and women, respectively.

Analysis of mortality rates according to the cause of death showed an odds ratio (OR) for overall death of 2.28 (95% CI, 2.18–2.39) in the neurodegenerative dementia group (Table 3). Patients with neurodegenerative dementia who died during this study had elevated ORs for mortality regardless of the cause of death except for those with neoplasms (false discovery rate-adjusted *P* < 0.05). Neurologic disease showed the highest OR for mortality (15.93; 95% CI, 13.10–19.38) followed by mental disease (4.89; 95% CI, 3.78–5.57). The causes of death are detailed in Supplementary Table 1.

## 4. Discussion

Our investigation of the Korean population revealed an elevated mortality rate among patients with neurodegenerative dementia across all age- and sex-based subgroups. The adjusted HR was highest in patients whose ages at the time of neurodegenerative dementia diagnosis were 60–69 years and lowest in those diagnosed at ages ≥ 80 years. Mortality rates owing to any of the investigated causes other than neoplasms were higher in the neurodegenerative dementia group than in the control group. Among the causes of death of patients with neurodegenerative dementia, neurologic disease showed the highest OR, followed by mental disease.

The adjusted overall HRs calculated in previous studies of mortality among patients with dementia were similar to ours [7, 11, 12, 18–20]; the causes of death of these patients were also similar [7, 11, 19] even though the number of studies was too few to enable the type of analysis we were able to perform in this study. However, our observation of adjusted HRs differing between age groups was not consistent with previous studies that performed the same analyses [7, 18, 21]. Previous studies varied in terms of patient ethnicity and age, size of study population, underlying causes of dementia (e.g., AD or vascular dementia), types of dementia (e.g., incidental or prevalent), follow-up duration, and statistical analysis methods [7, 12, 18–23]. Such differences might have caused some of the inconsistences between previously derived data and ours; the characteristics of the previous studies are summarized in Supplementary Table 2.

Our derived adjusted HR for mortality in our neurodegenerative dementia group was 2.11, which was consistent with previous studies of patients with this condition [7, 11, 12, 18–20]. A study by C. Wolfson et al. in Canada [18] and another by Ganguli et al. in the United States [19] found HRs for mortality (95% CIs) to be 1.52 (1.30–1.72) and 1.4 (1.2–1.8), respectively. A study by Villarejo et al. in Spain found that the HRs for mortality (95% CIs) were 2.23 (1.77–2.82), 3.10 (2.47–3.89), and 4.98 (3.85–6.44) in patients with mild, moderate, and severe dementia, respectively [7]. Previous studies in Korea revealed that patients with dementia had higher HRs for mortality even though their designs (particularly patient recruitment methods) varied [11, 12, 20]. One study found that the mortality rates of Koreans with dementia were 1.8–5.8-fold higher than that of the general Korean population, depending on age [11]. Another study of individuals with “cognitive impairment but no dementia” and of 69 individuals with clinically diagnosed dementia found that the HRs for mortality (95% CIs) were 1.92 (1.46–2.54) in the former group and 3.20 (2.30–4.44) in the latter [20]. A recent study of patients with prevalent and incident dementia at baseline assessment showed HRs for mortality (95% CIs) of 2.82 (1.28–6.22) and 8.37 (4.23–16.54), respectively [12]. The HR values found in these studies (including ours) were similar implying that advances in the treatment of neurodegenerative dementia have not necessarily improved mortality rates even though they may have contributed to improving the quality of life of patients with this condition. Alternatively, the mortality rates of patients with neurodegenerative dementia may indeed have improved, but the impact of this was diluted owing to the life expectancy of the general population increasing during the same period.

We observed the highest HR for mortality in patients who were diagnosed with neurodegenerative dementia at 60–69 years of age (3.39), followed by those who were diagnosed at 70–79 years (2.46) and those who were diagnosed at ≥80 years (1.77). Only a few investigators have analyzed the risk of mortality in different age groups, with a fraction reporting the HRs (most of which showed findings inconsistent with ours). C. Wolfson et al.'s study in Canada revealed HRs (95% CIs) of 2.36 (1.36–4.06), 4.26 (2.51–7.17), and 8.08 (4.39–12.94) for those aged 65–74, 75–84, and ≥85 years, respectively [18]. Villarejo et al.'s study in Spain revealed HRs (95% CIs) of 1.77 (1.57–1.99) and 2.67 (2.21–3.22) in those aged 75–84 and ≥85 years, respectively (the 65–74-year age group served as the baseline) [7]. A study in Sweden by Garcia-Ptacek et al. revealed HRs for mortality (95% CIs) of 1.96 (1.57–2.44), 3.32 (2.72–4.17), and 6.17 (4.97–7.65) among those aged 65–74, 75–84, and ≥85 years, respectively [21]. These data were consistent with the notion that AD development in relatively younger individuals poses a higher risk of mortality than it does among older counterparts [24, 25], as early-onset dementia has a more debilitating course. In contrast, a Swedish study by Agüero-Torres et al. found that the relative risks (95% CIs) were 3.3 (1.4–9.1) in men aged 77–84 years, 1.7 (0.8–3.5) in men ≥85 years, 4.5 (2.2–8.9) in women 77–84 years, and 2.4 (1.8–3.2) in women ≥85 years [22]; these values were similar to ours. These findings could be attributed to the fact that patients who are diagnosed at an older age are more likely to die of other causes, which dilutes the impact of neurodegenerative dementia on the mortality rate. The discrepancy might also have been a consequence of differences in study design, as mentioned above. Further studies are required to investigate this issue.

All disease categories except neoplasms were associated with an elevated OR for mortality in the dementia group; neurologic disease had the highest OR (15.93) followed by mental disease (4.89), implying that neurologic diseases that are likely to arise from dementia are associated with the highest mortality rates. Only a few studies investigated the causes of mortality in patients with dementia [7, 11, 19]; their findings were similar to ours. Ganguli et al.'s study in the United States found that the death certificates of individuals with AD were significantly more likely to list other brain disorders as the cause of death than were those of persons without dementia (5.5% vs. 1.7%). Dementia/AD was listed as the cause of death in 12.3% of individuals with AD and in 0.4% of those without dementia; conversely, the former patients were less likely to die of cancer than the latter (12.3% vs. 26.2%) [19]. Villarejo et al.'s study in Spain found that dementia itself and cerebrovascular disorders were significantly more common primary causes of death among patients with dementia than among control individuals (20.0% vs. 5.2% and 14.9% vs. 7.7%, respectively), whereas cancer was significantly less frequent in patients with dementia than in control individuals (5.8% vs. 26.5%) [7]. A Korean study by Go et al. found that the causes of death in patients with AD were (in order of frequency) dementia (36.0%), senescence (10.4%), stroke (9.6%), malignancy (9.1%), diabetes mellitus (6.3%), cardiovascular diseases (6.1%), and pneumonia (4.7%) [11], although they did not compare these frequencies to those occurring within the control population. Possible explanations for our own observations are that (i) the most common causes of death in the general population (such as cardiovascular disease) are also common in patients with dementia even though their frequencies per se are not much different than those in the general population and/or (ii) cancer (which is another common cause of death in the general population) might be underdiagnosed in those who are cognitively impaired [7]. Our results imply that caregivers should pay more attention to screening for other diseases, particularly cancer, in patients with neurodegenerative dementia while managing the dementia itself.

Our study had a few limitations. First, we did not distinguish between prevalent and incidental neurodegenerative dementia even though the risk of mortality between these 2 types differ. Second, we were unable to determine the severity of neurodegenerative dementia, which is a factor that reportedly influences mortality rates. Third, we included only diagnosed, symptomatic neurodegenerative dementia and were thus unable to evaluate the impact of mild cognitive impairment on mortality. Lastly, the death certificates only listed a single condition as the cause of death, thereby potentially ignoring other underlying ailments that may have contributed to mortality. Nevertheless, our observations are representative considering the scale of the data, and our findings ought to provide deeper insights into improving care for Korean patients with neurodegenerative dementia and ultimately contribute to decreasing mortality rates.

## 5. Conclusions

We performed the largest study on the risk of mortality among patients with neurodegenerative dementia in South Korea using clearly defined inclusion criteria and found that the risk of mortality increased in these patients regardless of age and sex. The HR was the highest in those who were diagnosed at 60–69 years of age, followed by those who were diagnosed at 70–79 years and was lowest among those who were diagnosed at ≥80 years. Death by any cause other than neoplasms—particularly neurologic disease and mental disease—showed an elevated risk in the neurodegenerative dementia group, while the risk of death owing to neoplasms in this group was lower.

## Figures and Tables

**Table 1:** General characteristics of the participants.

Characteristics	Total participants		
	Neurodegenerative dementia (*n*, %)	Control (*n*, %)	*P* value
Age (years)			1.000
60–64	580 (5.2)	2,320 (5.2)	
65–69	1,288 (11.5)	5,152 (11.5)	
70–74	2,321 (20.7)	9,284 (20.7)	
75–79	2,960 (26.4)	11,840 (26.4)	
80–84	2,588 (23.1)	10,352 (23.1)	
85+	1,478 (13.2)	5,912 (13.2)	
Sex			1.000
Male	3,568 (31.8)	14,272 (31.8)	
Female	7,647 (68.2)	30,588 (68.2)	
Income			1.000
1 (lowest)	1,280 (11.4)	5,120 (11.4)	
2	1,092 (9.7)	4,368 (9.7)	
3	447 (4.0)	1,788 (4.0)	
4	479 (4.3)	1,916 (4.3)	
5	519 (4.6)	2,076 (4.6)	
6	621 (5.5)	2,484 (5.5)	
7	711 (6.3)	2,844 (6.3)	
8	778 (6.9)	3,112 (6.9)	
9	1,067 (9.5)	4,268 (9.5)	
10	1,722 (15.4)	6,888 (15.4)	
11 (highest)	2,499 (22.3)	9,996 (22.3)	
Region of residence			1.000
Urban	4,493 (40.1)	17,972 (40.1)	
Rural	6,722 (59.9)	26,888 (59.9)	
Hypertension			1.000
Yes	8,200 (73.1)	32,800 (73.1)	
No	3,015 (26.9)	12,060 (26.9)	
Diabetes			1.000
Yes	3,942 (35.1)	15,768 (35.1)	
No	7,273 (64.9)	29,092 (64.9)	
Dyslipidemia			1.000
Yes	3,470 (30.9)	13,880 (30.9)	
No	7,745 (69.1)	30,980 (69.1)	
Ischemic heart disease			<0.001^∗^
Yes	1,670 (14.9)	5,884 (13.1)	
No	9,545 (85.1)	38,976 (86.9)	
Stroke			<0.001^∗^
Yes	5,425 (48.4)	11,356 (25.3)	
No	5,790 (51.6)	33,504 (74.7)	
Death			<0.001^∗^
Yes	3,873 (34.5)	8,434 (18.8)	
No	7,342 (65.5)	36,426 (81.2)	

^∗^Chi-square or Fisher's exact test. Significance set at a *P* value < 0.05.

**Table 2:** Crude and adjusted hazard ratios for mortality in patients with neurodegenerative dementia.

Characteristics	Hazard ratio (95% confidence interval)		
	Crude^†^	*P* value	Adjusted^†‡^	*P* value
Total participants (*n* = 56,075)				
Neurodegenerative dementia		<0.001^∗^		<0.001^∗^
Yes	2.26 (2.17–2.35)		2.11 (2.03–2.20)	
No	1.00		1.00	
Men (*n* = 17,840)				
Neurodegenerative dementia		<0.001^∗^		<0.001^∗^
Yes	2.59 (2.43–2.76)		2.41 (2.26–2.57)	
No	1.00		1.00	
Women (*n* = 38,235)				
Neurodegenerative dementia		<0.001^∗^		<0.001^∗^
Yes	2.09 (1.99–2.19)		1.96 (1.86–2.06)	
No	1.00		1.00	
Young (60–69 years, *n* = 9,340)				
Neurodegenerative dementia		<0.001^∗^		<0.001^∗^
Yes	3.81 (3.31–4.38)		3.25 (2.79–3.78)	
No	1.00		1.00	
Middle-aged (70–79 years, *n* = 26,405)				
Neurodegenerative dementia		<0.001^∗^		<0.001^∗^
Yes	2.77 (2.60–2.95)		2.48 (2.32–2.65)	
No	1.00		1.00	
Older (≥80 years, *n* = 20,330)				
Neurodegenerative dementia		<0.001^∗^		<0.001^∗^
Yes	1.84 (1.75–1.95)		1.78 (1.69–1.88)	
No	1.00		1.00	

^∗^Cox proportional hazards regression model. Significance set at a *P* value < 0.05.

^†^Stratified by age, sex, income, region of residence, hypertension, diabetes, and dyslipidemia.

^‡^A model adjusted for ischemic heart disease and stroke histories.

**Table 3:** Differences in mortality rates between patients in the neurodegenerative dementia and control groups according to the cause of death.

Cause of death	Total participants			
	Neurodegenerative dementia (*n* = 11,215)	Control (*n* = 44,860)	Odds ratio (95% CI)	*P* value
All causes of death (*n*, %)	3,873 (34.5)	8,434 (18.8)	2.28 (2.18–2.39)	<0.001^s^
Infection (*n*, %)	100 (0.9)	197 (0.4)	2.04 (1.60–2.60)	<0.001^∗^
Neoplasm (*n*, %)	408 (3.6)	1,846 (4.1)	0.88 (0.79–0.98)	0.021
Metabolic disease (*n*, %)	226 (2.0)	377 (0.8)	2.43 (2.06–2.87)	<0.001^∗^
Mental diseases (*n*, %)	219 (2.0)	194 (0.4)	4.89 (3.78–5.57)	<0.001^∗^
Neurologic disease (n, %)	489 (4.4)	128 (0.3)	15.93 (13.10–19.38)	<0.001^∗^
Circulatory disease (*n*, %)	1,123 (10.0)	2,329 (5.2)	2.03 (1.89–2.19)	<0.001^∗^
Respiratory disease (*n*, %)	441 (3.9)	812 (1.8)	2.22 (1.97–2.50)	<0.001^∗^
Digestive disease (*n*, %)	88 (0.8)	257 (0.6)	1.37 (1.08–1.75)	0.010^∗^
Muscular disease (*n*, %)	40 (0.4)	79 (0.2)	2.03 (1.39–2.97)	<0.001^∗^
Genitourinary disease (*n*, %)	88 (0.8)	167 (0.4)	2.12 (1.63–2.74)	<0.001^∗^
Trauma (*n*, %)	166 (1.5)	468 (1.0)	1.43 (1.19–1.70)	<0.001^∗^
Other (*n*, %)	485 (4.3)	1,580 (3.5)	1.24 (1.12–1.37)	<0.001^∗^

^∗^Chi-square or Fisher's exact test. Significance set at a false discovery rate-adjusted *P* value of < 0.05. CI: confidence interval.

## Data Availability

Release of the data by the researcher is not allowed legally. All of data are available from the database of National health Insurance Sharing Service (NHISS) https://nhiss.nhis.or.kr/.

## Supplementary Materials

Supplementary Materials Supplementary Table S1: the causes of death in patients with neurodegenerative dementia and their matched control groups. Supplementary Table S2: a comparison of studies that investigated the causes of death among patients with neurodegenerative dementia.
