# Supplementary material for: Causes of Mortality in Korean Patients with Neurodegenerative Dementia
Source: Biomed Res Int. 2022 Apr 25;2022:3206594. doi: 10.1155/2022/3206594 (PMC9060994; doi:10.1155/2022/3206594)
Supplement: Supplementary Materials — Supplementary Table S1: the causes of death in patients with neurodegenerative dementia and their matched control groups. Supplementary Table S2: a comparison of studies that investigated the causes of death among patients with neurodegenerative dementia. [file 3206594.f1.docx]

**Causes of Mortality in Korean Patients with Neurodegenerative Dementia**

Hyo Geun Choi^1,2^, Bumjung Park^2^, Ji Hee Kim^3^, Joo-Hee Kim^4^, Mi Jung Kwon^5^, and Miyoung Kim^6^*

^1^Hallym Data Science Laboratory, Hallym University College of Medicine, Anyang, South Korea

^2^Department of Otorhinolaryngology-Head & Neck Surgery, Hallym University Sacred Heart Hospital, Hallym University College of Medicine, Anyang, South Korea

^3^Department of Neurosurgery, Hallym University Sacred Heart Hospital, Hallym University College of Medicine, Anyang, South Korea

^4^Division of Pulmonary, Allergy, and Critical Care medicine, Department of Medicine, Hallym University Sacred Heart Hospital, Hallym University College of Medicine, Anyang, South Korea

^5^Department of Pathology, Hallym University Sacred Heart Hospital, Hallym University College of Medicine, Anyang, South Korea

^6^Department of Laboratory Medicine, Asan Medical Center, University of Ulsan College of Medicine, 88 Olympic-ro 43-gil, Songpa-gu, Seoul 05505, South Korea

**S1 Table** Causes of death among patients in the neurodegenerative dementia and control groups

| Cause of death | | Codes | Number of participants | |
| --- | --- | --- | --- | --- |
|  | |  | Neurodegenerative dementia  (n = 11,215) | Control  ( n = 44,860) |
| **Infection** | | A00–B99 |  |  |
|  | Intestinal infectious diseases | A00–A09 | 15 | 25 |
|  | Tuberculosis | A15–A19 | 35 | 73 |
|  | Certain zoonotic bacterial diseases | A20–A28 | 0 | 0 |
|  | Other bacterial diseases | A30– A49 | 37 | 73 |
|  | Infections with a predominantly sexual mode of transmission | A50–A64 | 1 | 0 |
|  | Other spirochetal diseases | A65–A69 | 0 | 0 |
|  | Other diseases caused by chlamydia | A70–A74 | 0 | 0 |
|  | Rickettsioses | A75–A79 | 1 | 4 |
|  | Viral infections of the central nervous system | A80–A89 | 1 | 1 |
|  | Arthropod-borne viral fevers and viral hemorrhagic fevers | A92–A99 | 0 | 1 |
|  | Viral infections characterized by skin and mucous membrane lesions | B00–B09 | 0 | 2 |
|  | Viral hepatitis | B15–B19 | 7 | 13 |
|  | Acquired immunodeficiency syndrome | B20–B24 | 0 | 0 |
|  | Other viral diseases | B25–B34 | 0 | 0 |
|  | Mycoses | B35–B49 | 1 | 2 |
|  | Protozoal diseases | B50–B64 | 0 | 0 |
|  | Helminthiases | B65–B83 | 0 | 0 |
|  | Pediculosis, acariasis, and other infestations | B85–B89 | 0 | 0 |
|  | Sequelae of infectious and parasitic diseases | B90–B94 | 2 | 3 |
|  | Bacterial, viral, and other infectious agents | B95–B98 | 0 | 0 |
|  | Other infectious diseases | B99 | 0 | 0 |
| **Neoplasm** | | C00–D48 |  |  |
|  | Malignant neoplasm of the lip, oral cavity, and pharynx | C00–C14 | 5 | 13 |
|  | Malignant neoplasms of digestive organs | C15–C26 | 190 | 991 |
|  | Malignant neoplasms of respiratory and intrathoracic organs | C30–C39 | 98 | 406 |
|  | Malignant neoplasm of the bone and articular cartilage | C40–C41 | 0 | 3 |
|  | Melanoma and other malignant neoplasms of the skin | C43–C44 | 9 | 12 |
|  | Malignant neoplasms of mesothelial and soft tissues | C45–C49 | 3 | 10 |
|  | Malignant neoplasm of the breast | C50 | 6 | 16 |
|  | Malignant neoplasm of female genital organs | C51–C58 | 11 | 62 |
|  | Malignant neoplasm of male genital organs | C60–C63 | 16 | 71 |
|  | Malignant neoplasm of the urinary tract | C64–C68 | 21 | 70 |
|  | Malignant neoplasm of the eye, brain, and other parts of central nervous system | C69–C72 | 11 | 16 |
|  | Malignant neoplasm of the thyroid and other endocrine glands | C73–C75 | 4 | 19 |
|  | Malignant neoplasm of ill-defined, secondary, and unspecified sites | C76–C80 | 10 | 30 |
|  | Malignant neoplasms of lymphoid, hematopoietic, and related tissues | C81–C96 | 11 | 83 |
|  | Malignant neoplasm of independent (primary) multiple sites | C97 | 2 | 4 |
|  | In situ neoplasms | D00–D09 | 0 | 0 |
|  | Benign neoplasms | D10–D36 | 2 | 7 |
|  | Neoplasms of uncertain or unknown behavior | D37–D48 | 9 | 33 |
| **Metabolic disease** | | E00–E90 |  |  |
|  | Disorders of the thyroid gland | E00–E07 | 1 | 2 |
|  | Diabetes mellitus | E10–E14 | 216 | 359 |
|  | Other disorders of glucose regulation and pancreatic internal secretion | E15–E16 | 1 | 0 |
|  | Disorders of other endocrine glands | E20–E35 | 1 | 2 |
|  | Malnutrition | E40–E46 | 1 | 3 |
|  | Other nutritional deficiencies | E50–E64 | 1 | 3 |
|  | Obesity and other hyperalimentation | E65–E68 | 0 | 0 |
|  | Metabolic disorders | E70–E90 | 5 | 8 |
| **Mental disease** | | F00–F99 | 219 | 194 |
|  | Organic, including symptomatic mental disorders | F00–F09 | 215 | 187 |
|  | Mental and behavioral disorders due to psychoactive substance use | F10–F19 | 3 | 5 |
|  | Schizophrenia, schizotypal, and delusional disorders | F20–F29 | 0 | 2 |
|  | Mood affective disorders | F30–F39 | 0 | 0 |
|  | Neurotic, stress-related, and somatoform disorders | F40–F48 | 1 | 0 |
|  | Behavioral syndromes associated with physiological disturbances and physical factors | F50–F59 | 0 | 0 |
|  | Disorders of adult personality and behavior | F60–F69 | 0 | 0 |
|  | Mental retardation | F70–F79 | 0 | 0 |
|  | Disorders of psychological development | F80–F89 | 0 | 0 |
|  | Behavioral and emotional disorders with onset usually occurring in childhood and adolescence | F90–F98 | 0 | 0 |
|  | Unspecified mental disorder | F99 | 0 | 0 |
| **Neurologic disease** | | G00–G99 |  |  |
|  | Inflammatory diseases of the central nervous system | G00–G09 | 2 | 2 |
|  | Systemic atrophies primarily affecting the central nervous system | G10–G14 | 4 | 6 |
|  | Extrapyramidal and movement disorders | G20–G26 | 116 | 69 |
|  | Other degenerative diseases of the nervous system | G30–G32 | 352 | 22 |
|  | Demyelinating diseases of the central nervous system | G35–G37 | 0 | 0 |
|  | Episodic and paroxysmal disorders | G40–G47 | 7 | 8 |
|  | Nerve, nerve root, and plexus disorders | G50–G59 | 0 | 0 |
|  | Polyneuropathies and other disorders of the peripheral nervous system | G60–G64 | 0 | 0 |
|  | Diseases of myoneural junction and muscle | G70–G73 | 1 | 3 |
|  | Cerebral palsy and other paralytic syndromes | G80–G83 | 0 | 4 |
|  | Other disorders of the nervous system | G90–G99 | 7 | 14 |
| **Circulatory disease** | | I00–I99 |  |  |
|  | Acute rheumatic fever | I00–I02 | 0 | 0 |
|  | Chronic rheumatic heart diseases | I05–I09 | 3 | 4 |
|  | Hypertensive diseases | I10–I15 | 154 | 269 |
|  | Ischemic heart diseases | I20–I25 | 209 | 505 |
|  | Pulmonary heart disease and diseases of pulmonary circulation | I26–I28 | 2 | 16 |
|  | Other forms of heart disease | I30–I52 | 154 | 424 |
|  | Cerebrovascular diseases | I60–I69 | 579 | 1074 |
|  | Diseases of the arteries, arterioles, and capillaries | I70–I79 | 17 | 35 |
|  | Diseases of the veins, lymphatic vessels, and lymph nodes, and necrotizing enterocolitis | I80–I89 | 4 | 2 |
|  | Other and unspecified disorders of the circulatory system | I95–I99 | 1 | 0 |
| **Respiratory disease** | | J00–J99 |  |  |
|  | Acute upper respiratory infections | J00–J06 | 0 | 2 |
|  | Influenza and pneumonia | J09–J18 | 240 | 338 |
|  | Other acute lower respiratory infections | J20–J22 | 0 | 1 |
|  | Other diseases of upper respiratory tract | J30–J39 | 1 | 1 |
|  | Chronic lower respiratory diseases | J40–J47 | 126 | 333 |
|  | Lung diseases due to external agents | J60–J70 | 49 | 47 |
|  | Other respiratory diseases principally affecting the interstitium | J80–J84 | 13 | 49 |
|  | Suppurative and necrotic conditions of lower respiratory tract | J85–J86 | 3 | 7 |
|  | Other diseases of pleura | J90–J94 | 1 | 6 |
|  | Other diseases of the respiratory system | J95–J99 | 8 | 28 |
| **Digestive disease** | | K00–K93 |  |  |
|  | Diseases of the oral cavity, salivary glands, and jaws | K00–K14 | 0 | 0 |
|  | Diseases of the esophagus, stomach, and duodenum | K20–K31 | 15 | 34 |
|  | Diseases of the appendix | K35–K38 | 0 | 1 |
|  | Hernia | K40–K46 | 1 | 2 |
|  | Noninfective enteritis and colitis | K50–K52 | 1 | 3 |
|  | Other diseases of the intestines | K55–K64 | 14 | 51 |
|  | Diseases of the peritoneum | K65–K67 | 4 | 8 |
|  | Diseases of the liver | K70–K77 | 23 | 84 |
|  | Disorders of the gallbladder, biliary tract, and pancreas | K80–K87 | 17 | 49 |
|  | Other diseases of the digestive system | K90–K93 | 13 | 25 |
| **Muscular disease** | | M00–M99 |  |  |
|  | Infectious arthropathies | M00–M03 | 3 | 2 |
|  | Inflammatory polyarthropathies | M05–M14 | 7 | 9 |
|  | Arthrosis | M15–M19 | 5 | 7 |
|  | Other joint disorders | M20–M25 | 2 | 0 |
|  | Systemic connective tissue disorder | M30–M36 | 0 | 4 |
|  | Deforming dorsopathies | M40–M43 | 0 | 1 |
|  | Spondylopathies | M45–M49 | 3 | 8 |
|  | Other dorsopathies | M50–M54 | 1 | 3 |
|  | Disorders of the muscles | M60–M63 | 0 | 3 |
|  | Disorders of the synovium and tendon | M65–M68 | 0 | 0 |
|  | Other soft tissue disorders | M70–M79 | 1 | 4 |
|  | Disorders of bone density and structure | M80–M85 | 15 | 38 |
|  | Other osteopathies | M86–M90 | 3 | 0 |
|  | Chondropathies | M91–M94 | 0 | 0 |
|  | Other disorders of the musculoskeletal system and connective tissue | M95–M99 | 0 | 0 |
| **Genitourinary disease** | | N00–N99 |  |  |
|  | Glomerular diseases | N00–N08 | 1 | 7 |
|  | Renal tubulo-interstitial diseases | N10–N16 | 3 | 11 |
|  | Renal failure | N17–N19 | 69 | 116 |
|  | Urolithiasis | N20–N23 | 1 | 1 |
|  | Other disorders of the kidney and ureter | N25–N29 | 0 | 2 |
|  | Other diseases of the urinary system | N30–N39 | 13 | 27 |
|  | Diseases of male genital organs | N40–N51 | 1 | 3 |
|  | Disorders of the breast | N60–N64 | 0 | 0 |
|  | Inflammatory diseases of female pelvic organs | N70–N77 | 0 | 0 |
|  | Noninflammatory disorders of the female genital tract | N80–N98 | 0 | 0 |
|  | Other disorders of the genitourinary tract | N99 | 0 | 0 |
| **Abnormal finding** | | R00–R99 |  |  |
|  | Symptoms and signs involving the circulatory and respiratory systems | R00–R09 | 31 | 52 |
|  | Symptoms and signs involving the digestive system and abdomen | R10–R19 | 0 | 1 |
|  | Symptoms and signs involving the skin and subcutaneous tissue | R20–R23 | 0 | 0 |
|  | Symptoms and signs involving the nervous and musculoskeletal systems | R25–R29 | 0 | 0 |
|  | Symptoms and signs involving the urinary system | R30–R39 | 0 | 0 |
|  | Symptoms and signs involving cognition, perception, emotional state, and behavior | R40–R46 | 0 | 0 |
|  | Symptoms and signs involving speech and voice | R47–R49 | 0 | 0 |
|  | General symptoms and signs | R50–R69 | 350 | 1276 |
|  | Abnormal findings on examination of blood, without diagnosis | R70–R79 | 0 | 0 |
|  | Abnormal findings on examination of urine, without diagnosis | R80–R82 | 0 | 0 |
|  | Abnormal findings on examination of other body fluids, substances, and tissues, without diagnosis | R83–R89 | 0 | 0 |
|  | Abnormal findings on diagnostic imaging and in function studies, without diagnosis | R90–R94 | 0 | 0 |
|  | Ill-defined and unknown causes of mortality | R95–R99 | 40 | 112 |
| **Trauma** | | S00–T98 |  |  |
|  | Injuries to the head | S00–S09 | 23 | 84 |
|  | Injuries to the neck | S10–S19 | 2 | 0 |
|  | Injuries to the thorax | S20–S29 | 3 | 14 |
|  | Injuries to the abdomen, lower back, lumbar spine, and pelvis | S30–S39 | 3 | 22 |
|  | Injuries to the shoulder and upper arm | S40–S49 | 1 | 2 |
|  | Injuries to the elbow and forearm | S50–S59 | 0 | 3 |
|  | Injuries to the wrist and hand | S60–S69 | 0 | 2 |
|  | Injuries to the hip and thigh | S70–S79 | 45 | 74 |
|  | Injuries to the knee and lower leg | S80–S89 | 0 | 2 |
|  | Injuries to the ankle and foot | S90–S99 | 0 | 0 |
|  | Injuries involving multiple body regions | T00–T07 | 18 | 38 |
|  | Injuries to unspecified part of the trunk, limb, or body region | T08–T14 | 7 | 24 |
|  | Effects of foreign body entering through a natural orifice | T15–T19 | 11 | 12 |
|  | Burns and corrosions of the external body surface, specified by site | T20–T25 | 0 | 1 |
|  | Burn and corrosions confined to eye and internal organs | T26–T28 | 0 | 1 |
|  | Burns and corrosions of multiple and unspecified body regions | T29–T32 | 2 | 3 |
|  | Frostbite | T33–T35 | 0 | 0 |
|  | Poisoning by drugs, medicaments, and biological substances | T36–T50 | 0 | 3 |
|  | Toxic effects of substances chiefly nonmedicinal as to source | T51–T65 | 20 | 79 |
|  | Other and unspecified effects of external causes | T66–T78 | 20 | 88 |
|  | Certain early complications of trauma | T79 | 1 | 3 |
|  | Complications of surgical and medical care, necrotizing enterocolitis | T80–T88 | 0 | 2 |
|  | Sequelae of injures, poisoning, and other consequences of external causes | T90–T98 | 10 | 11 |
| **Others** | | D50–D89, L00–L99 |  |  |
|  | Nutritional anemias | D50–D53 | 2 | 4 |
|  | Hemolytic anemias | D55–D59 | 0 | 1 |
|  | Aplastic and other anemias | D60–D64 | 2 | 11 |
|  | Coagulation defect, purpura, and other hemorrhage conditions | D65–D69 | 1 | 6 |
|  | Other disease of blood and blood-forming organs | D70–D77 | 0 | 4 |
|  | Certain disorders involving the immune mechanism | D80–D89 | 1 | 0 |
|  | Other disorders of the skin and subcutaneous tissue | L80–L99 | 12 | 5 |
|  | Missing information |  | 46 | 13 |

**S2 Table** Comparison of studies performed to date.

|  |  |  |  |  |  |  |  |  |  |  |
| --- | --- | --- | --- | --- | --- | --- | --- | --- | --- | --- |
| Reference Number | Country | Published Year | Recruitment period | Number of patients | Study design | Disease category | Subcategorization | Age group | Follow-up period | Cause of death |
| Current study | Korea | - | 2002–2013 | 11,215 | Cohort study (screening of 1,125,691 subjects) | AD* | - | 60–69, 70–79, and ≥80 years | Median 41.9 months (SD† = 32.4 months), up to 12 years | Included |
| 7 | Spain | 2011 | 1994–1995 | 306 | Cohort study (screening of 5,278 subjects) | AD vs. VaD‡ | Mild, moderate, and severe dementia | 65–74, 75–84, and ≥85 years | Up to 13 years | Included |
| 8 | United States | 2007 | 1992–1994; 1999–2002 | 192 | Case series (screening of 4307 community-based individuals) | AD | Mediterranean diet | ≥65 years | Mean 4.4 years (SD = 3.6 years, range 0.2–13.6 years) | Not included |
| 11 | Korea | 2013 | 1995–2005 | 724 | Case series from a single hospital | AD | - | ≤59, 60–64, 65–69, 70–74, 75–79, and ≥80 years | Mean 10.2 years (SD = 3.8 years) from onset and 7.3 years (SD = 3.3 years) from diagnosis | Included |
| 12 | Korea | 2019 | 2010–2012 | 334 | Cohort study (screening of 6,752 subjects) | Mild cognitive impairment, AD vs. dementia | Prevalent dementia vs. incident dementia | ≥60 years | 2 years | Not included |
| 18 | Canada | 2001 | 1996–1997 | 821 | Cohort study (screening of 10,263 subjects) | Probable AD vs. possible AD vs. VaD | - | <65, 65–74, 75–84, and ≥85 years | Up to 400 months | Not included |
| 19 | United States | 2005 | 1987–2002 | 330 | Cohort study (screening of 1,670 subjects) | AD | - | <65–74, 75–84, and ≥85 years | Mean 10.3 years (SD = 10.3 years, range 0.02–15.8 years) | Included |
| 20 | Sweden | 2014 | 2008–2011 | 15,209 | Cohort study | Dementia | AD, frontotemporal dementia, VaD, Lewy body dementia, and Parkinson’s disease-related dementia | 45–54, 55–64, 65–74, 75–84, and ≥85 years | Mean 2.5 years (range 0–1869 days) | Not included |
| 21 | Sweden | 1999 | 1987; 1990–1992 | 127 | Cohort study (screening of 917 subjects) | AD vs. VaD | - | 77–84 and ≥85 years | Up to 5 years | Included |
| 22 | United States | 2005 | 2002 | 956 | Case series from a single hospital | Dementia | All dementia vs. AD | <70, 70–79 and ≥80 years | Not specified | Not included |
| 23 | Korea | 2014 | 1996–1997 | 224 | Cohort study (screening of 1,035 patients at risk) | Cognitive impairment but no dementia vs. clinically diagnosed dementia | Cognitive impairment but no dementia vs. clinically diagnosed dementia (AD vs. VaD) | ≥65 years | Up to 8 years | Not included |

*AD: Alzheimer's disease; †SD: Standard deviation; ‡VaD: Vascular dementia
